# Allogeneic Hematopoietic Cell Transplantation in the Treatment of Chronic Lymphocytic Leukemia: Why and When?

**DOI:** 10.4084/MJHID.2010.018

**Published:** 2010-07-10

**Authors:** Maria L. Delioukina, Stephen J. Forman

**Affiliations:** Division of Hematology/Hematopoietic Cell Transplantation, City of Hope, Duarte, CA, USA

## Introduction:

Chronic lymphocytic leukemia (CLL) is the most common hematologic malignancy in adults with an incidence rate of 4.2 per 100,000 per year. CLL frequently takes an indolent course, with some patients not requiring treatment for years, yet is incurable by currently available chemo- and immuno-therapeutic modalities. Despite high initial response rates, particularly to purine analogues, patients invariably relapse and subsequently develop resistance to therapy. The traditional “watchful waiting” approach to CLL is being challenged by data showing that treatments used early in the disease course impact long-term overall and progression-free survivals.^1^–^2^ The only curative treatment for CLL currently, is allogeneic hematopoeietic cell transplantation (alloHCT).

In contrast to autologous transplant, myeloablative alloHCT for CLL patients generates durable remissions with promising survival plateaus; however, significant transplant related mortality (TRM) is also observed (25–50%).^3^–^4^ Rather than conditioning intensity, the graft-versus-leukemia (GVL) effects appears to be the primary mechanism behind long-term remission in this patient population,^5^–^6^ with some patients achieving CR after many months delay.^4^ The evidence supporting the role of GVL is based on studies showing decreased risk of relapse in patients with chronic GVHD, increased risk of relapse with T-cell depletion, remission generated by donor lymphocyte infusion, as well as gradual elimination of minimal residual disease.^4^,^6^–^8^

As the average age of CLL patients at diagnosis is 72 years, reduced intensity conditioning (RIC) regimens are frequently necessary to decrease TRM and increase the availability of alloHCT for CLL.^9^–^11^ Due to this age skew, combined with the fact that GVL dominates the curative process, the majority of data on alloHCT for CLL has been performed using reduced intensity regimens. Despite the lack of head-to-head comparisons between RIC and myeloablative regimens, RIC has become standard in the field

The selection of CLL patients for transplant is generally based on the European Group for Blood and Marrow Transplantation (EBMT) 2007 guidelines.^12^ According to the EBMT CLL transplant consensus one of several criteria must be fulfilled for the prognostic risk to justify alloHCT. The first category of poor-risk criteria involves disease that is refractory to purine analogues; this includes primary refractory disease and recurrence within short intervals following initial treatment with purine analogue combination therapy. The highest-risk category includes patients with del(17p13) or other TP53 gene mutations.

In the absence of multi-center randomized prospective studies, the biggest challenges in CLL alloHCT therapy include selection of patients and timing of transplant during the disease course. To illustrate our approach to alloHCT for CLL patients, several case scenarios are presented, each followed by a discussion of the therapeutic implications.

## Case Discussions

### Patient 1: Very high-risk features del(17p13)

A 45-year-old man presented with B-cell CLL, stage I Rai. His white blood cell count at presentation was 120 x 10^9^/L with 95% lymphocytosis, bulky cervical lymphadenopathy and constitutional symptoms of fatigue and night sweats. Cytogenetic analysis of the bone marrow using FISH studies showed del(17p13). The patient was treated with standard first-line therapy, fludarabine / cyclophosphamide / rituximab (FCR), and achieved a complete clinical response with normalization of peripheral blood count and lymph node size. He had an HLA-matched sibling. Based on the EBMT consensus, alloHCT would be recommended for such a patient.

As multiple prognostic factors for CLL are being investigated, determining which are the most useful for assessing candidacy for alloHCT has been challenging. The four molecular biological features that have the best track records for use as markers of aggressive disease and clinical prognostic parameters are: recurrent cytogenetic abnormalities as identified by FISH testing,^8^,^13^–^14^ immunoglobulin variable heavy-chain (IGVH) mutational status,^15^–^16^ p53 gene deletion,^17^–^18^ ZAP 70 expression,^19^–^20^ and CD38 protein expression.^16^

Cytogenetic abnormalities as assessed by FISH are a widely-applied prognostic tool. In an analysis by Dohner *et al.*,^13^ patients with deletions in chromosome band 17p13 had a median survival from diagnosis of 2–3 years as compared to 6–7 years for those with deletions in 11q22, and 9 years for those with a normal karyotype. In addition to their ability to predict treatment-free survival and OS, some biomarkers are also useful for predicting response to specific therapeutic agents. The presence of del(17p) and/or abnormal p53 function have consistently been shown to identify CLL patients whose responses to purine nucleoside analogs and alkylating agents are short-lived.^13^–^14^,^17^–^18^ Patients with 17p deletions experience treatment-free intervals of only 9 months and PFS intervals of 11 months after fludarabine based chemotherapy.^13^–^14^ The molecular basis for this clinical observation is disruption of p53-dependent apoptosis, responsible for the anti-leukemic effect of purine analogs and alkylating agents, in patients with del(17p13).

The weight of evidence does not convincingly demonstrate that IGHV mutational status predicts progression-free survival after transplant, with studies by Byrd *et al.*^17^ and Grever *et al.*^14^ obtaining conflicting results. The ability of ZAP 70 and CD38 expression to predict response to treatment and PFS/PS after treatment is even less clear.^16^,^19^–^21^

Multiple studies demonstrate that allogeneic transplant can overcome the detrimental effects of negative indicators such as 17p and 11q deletion on prognosis^5^–^6^,^22^ and City of Hope experience also confirms this observation. This leveling of the playing field is particularly important, as no other options apart from clinical trials are available for these patients after they fail standard chemotherapy regimens.

Appropriate patients with del(17p13) are candidates for allogeneic stem cell transplant early in the course of the disease – 1^st^ or 2^nd^ remission, given their high risk of non response for first-line therapy and/or short duration of response. In this regard it is reasonable to initiate a donor search at the time first-line therapy is initiated for patients with a 17p13 deletion.

### Patient 2: Poor-risk features (progression <24month)

A 62 year old woman was diagnosed with B-cell CLL manifested as peripheral lymphocytosis only. After 3 years of a watchful waiting approach, treatment was initiated based on peripheral lymphocytosis and constitutional symptoms. FISH cytogenetic testing was performed on peripheral blood and no 17p or 11q abnormalities were identified. She was treated with FCR as standard first-line treatment and achieved clinical remission with normalization of peripheral lymphocytosis and resolution of B symptoms. Within 21 months she progressed, developing cervical and axillary lymphadenopathy and recurrent lymphocytosis, and was subsequently treated with bendamustin and rituxamab. The patient was only able to tolerate an abbreviated number of treatment courses due to pancytopenia and achieved a partial response. The FISH cytogenetic testing was repeated prior to second-line treatment and showed a new clone with an 11q deletion. The patient had no HLA-matched sibling but a 10/10 matched unrelated donor was identified at that time.

We recommend repeat testing for high-risk features at the time of each relapse or progression event due to the evolving biology of this disease and the acquired nature of these mutations. New cytogenetic abnormalities are acquired during follow-up in more that 25% of patients over a 5 year interval^23^ and are associated with short survival. If a patient acquires poor genetic features during the course of the disease or progresses within 24 months after initial therapy, the indication for alloHCT becomes more relevant compared to the standard choice of second and third line treatment.

This patient’s 2- month progression in combination with the acquired cytogenetic abnormality made her a candidate for allo HCT. The use of reduced intensity conditioning has become standard for patients with CLL and decreases transplant-associated toxicities, especially for older patients. The effectiveness of reduced-intensity HCT for the treatment of CLL has been reported in the literature since 2003. Sorror *et al*. report that 64 patients treated with a non-myeloablative protocol using low dose TBI have a 2-year OS of 60%, DFS of 52% with a TRM 22% and significant GVHD.^11^ Using a non-myeloabative FCR conditioning regimen employing early tapering of immunosupression and use of rituxan and DLI for immunomodulation, Khouri *et al.* estimate a 4-year OS of 48% and a current PFS of 44%.^5^ In 46 patients treated using a non-myeloablative regimen of fludarabine and low dose busulfan, Brown *et al.* report a 2-year OS of 54% and PFS of 34% with 17% TRM and a 2-year cumulative incidence of relapse of 48%.^24^

Our own data from COH were presented at the Rome Congress, “New drugs and hematopoietic stem cell transplantation in oncohematological diseases of the elderly” as an oral presentation in November of 2009.^25^ We presented an analysis of data from 27 CLL patients treated using alloHCT with fludarabine-based reduced intensity conditioning demonstrate overall survivals (OS) and progression-free survivals (PFS) of 80.0% and 72.8% at 1 year and 64.0% and 62.4% at 2 years. The relapse/progression rate was 15.4% and the non-relapse mortality (NRM) was 24.7%, at 2 years. The best response post transplant was complete remission in 19 patients (70.4%), partial response in 4 (14.8%) and stable disease in one (3.7%).^25^

For patients such as this man, who do not have HLA-matched siblings, use of a matched unrelated donor (MUD) is a valuable option. Based on extensive registry data from the EBMT,^26^ the Center for International Blood and Marrow Transplant Research (CIBMTR)^27^ and multivariate analysis of data effecting OS after RIC and myeloablative HCT in patients with CLL, there is no evidence of the inferiority of a well-matched MUD (10/10) versus HLA-matched sibling donors. Therefore, for older CLL patients with good performance status MUD transplant with reduced intensity conditioning would be a reasonable choice with curative intent.

### Patient 3: Disease refractory to fludarabine and alemtuzumab

A 55-year-old male patient with B-cell CLL, diagnosed initially at stage I Rai, manifested with mild lymphocytosis and small peripheral lymphadenopathy. The patient was observed for 5 years and eventually developed bulky lymphadenopahy, and was treated with FCR, resulting in a complete response. After 3 years, he developed recurrent lymphocytosis and lymphadenopathy, and was re-treated with fludarabine/rituximab with no response and significant cytopenia. He was subsequently treated with alemtuzumab, attaining partial response. The patient progressed again within 1 year, and was treated with a bendamustin and rituxan combination with no response. The patient had an HLA-matched sibling.

Unfortunately, this is an all-too-familiar scenario with CLL patients going to transplant. They have often been heavily pre-treated and eventually become chemo-resistant. Ruling out transformation into more aggressive types of lymphoma, known as Richter’s syndrome, is an important step in the assessment of CLL patients, particularly when confronted with progressively refractory behavior. Richter’s transformation diagnosed in a CLL patient at any time during the disease course is by itself an indication for allogeneic stem cell transplant as these patients have a dismal prognosis.^28^

At COH we have found that chemotherapy-refractory disease is associated with inferior progression-free survival in our RIC allo HCT patients, an association supported by Sorror *et al.*^29^ in a study with a large percentage of chemoresistant patients. Bulky lymphadenopathy at the time of transplant is also associated with poor progression-free survival in our patient population.^25^ It is possible that chemo-resistance could be a marker of aggressive disease that progresses too rapidly to be controlled by even an active immune response of GVL rather that actual predictor of intrinsic resistance to GVL activity. Therefore, would cytoreduction before transplantation improve outcome?

The use of non-myeloablative conditioning could possibly result in insufficient cytoreduction, which would interfere with engraftment and the anti-leukemia effect by not allowing time for development of GVL. More aggressive cytoreduction approaches may be necessary to enhance the GVL effect. Aggressive debulking prior to transplant, the use of reduced intensity regimens (such as fludarabine/melphalan) rather than purely non-ablative preparative regimens (such as FCR or single-dose TBI-based), and additional immunotherapy with monoclonal antibodies during or following transplant, are potential ways to improve the efficacy of transplant with reduced intensity conditioning.

Aggressive debulking prior to transplant is advocated by MD Anderson with a chemotherapy regimen such as oxaliplatin/fludarabine/cytarabine/rituximab (OFAR). A total of 70% of the patients treated with this approach achieve prolonged survival after subsequent HCT.^30^ Addition of the monoclonal antibody alemtuzumab can decrease GVHD while contributing to disease control, but this delays post-HCT immune reconstitution, increases the risk of infectious complications and impairs the GVL effect. Delgado *et al.* report on 41 patients treated with fludarabine, melphalan and alemtuzumab demonstrating an OS at 2 years of 51% and a relapse risk of 29% at 2 years; TRM is also 29% due to a high incidence of fungal and vial infections.^31^

The addition of high dose rituximab to pre-transplant conditioning has been employed^10^,^32^ and may serve several purposes. The most obvious is bone marrow cytoreduction to allow time for the GVL effect. Inclusion of rituximab as part of the preparative regimen would act to deplete both recipient and donor derived B-cells. There is also evidence that B-cells functioning as antigen-presenting cells may have an important role in the pathogenesis of GVHD,^33^ so rituximab could also lower the incidence of GVHD via their elimination.

It is preferable to refer patients for transplant before they become truly refractory to chemotherapy, as patients with transformed or bulky disease at the time of transplant have inferior outcomes. In addition, protracted treatment with nucleoside analogs and monoclonal antibodies, both T- and B-cell depleting, can contribute to immunosuppression and increase the risk of opportunistic infections during transplant or salvage chemotherapy. However, for a patient such as this, debulking could be attempted with a regimen such as oxaliplatin/fludarabine/cytarabine/rituximab (OFAR) or salvage regimens employed for agressive B-cell lymphomas. Response to salvage treatment prior to transplant would correlate with improved transplant outcome. In terms of conditioning regimen, the choice of reduced intensity but still cytoreductive conditioning with addition of anti CD20 monoclonal antibody rather than purely non myelobalative would be optimal. The transplantation-related risk, including major infectious episodes needs to be emphasized during and after transplantation.

### Patient 4: Delayed achievement of CR assessed by MRD

A 65 year-old patient was initially diagnosed with peripheral lymphocytosis. After 2 years of observation he progressed with peripheral lymphocytosis and was treated with FCR. No cytogenetic abnormalities were found on peripheral blood FISH studies. He remained in remission for 3 years and eventually developed progression of lymphocytosis with no significant lymph node involvement and was treated with alemtuzumab, achieving partial resposne. After one year he developed pancytopenia with 70% leukemia involvement of the bone marrow. He was unable to tolerate bendamustin/rituxan due to cytopenia. The patient was found to have an HLA-matched sibling and underwent non myeloablative alloHCT with fludarabine / cyclophosphamide / rituximab conditioning with gross bone marrow involvement at the time of transplant. He tolerated the conditioning well, and achieved 90% engraftment by STR analysis but showed evidence of residual disease by positive MRD testing at day 100. He was treated pre-emptively with rituximab and achieved MRD negativity at 6 months post transplant.

The quality of remission represented by cytogenetic, molecular and minimal residual disease (MRD) assays is being increasingly recognized as an important prognostic factor in many hematologic malignancies. As the goal in CLL treatment has moved from palliative care toward durable remission, MRD monitoring has become a significant assessment tool in the management of CLL. MRD eradication is an important target in the treatment of CLL because MRD negativity is clearly correlated with improved outcome.^4^–^6^,^34^ With standard chemotherapy regimens disease progression is inevitable in patients who are MRD-positive, whereas MRD-negative patients are able to attain durable remissions.^35^

Studies of unmutated IgVH MRD kinetics following autologous and allogeneic transplant^6^,^36^ showed that the presence of detectable unmutated IgVH MRD early after transplant does not have an impact on allograft outcome in CLL. Ritgen *et al.* report that MRD becomes undetectable beyond day 100 in 78% patients after alloHCT and correlates with long-term CR and MR. On the other hand, negative MRD states achieved after autoHCT with similar conditioning are not durable and the presence of detectable MRD post autoHCT is associated with high risk for relapse. These observations suggest that the GVL effect is the driving force behind clearance of MRD after transplant.

Patients with a high tumor burden or atypical morphology (Richters transformation) are at highest risk for early progression after alloHCT and are good candidates for MRD testing after alloHCT. The use of MRD allows post-transplant detection of preclinical relapse, enabling the early initiation of adoptive immunotherapy including prompt withdrawal of immunosupression, donor lymphocyte infusion (DLI), monoclonal antibody infusion, or a combination of immunotherapies. Khouri *et al*: report on a non-myeloabative FCR conditioning regimen employing early tapering of immunosupression and use of rituxan and DLI for immunomodulation.^5^ In this study, progression-free survival, prior to any immunomodulatory treatment was 30% at 5 years, but when including the immunomodulatory treatment of persistent or progressive disease as part of the regimen, “current progression-free survival” attained 53% at five years.

MRD assessment is performed using standardized protocols of either 4-color flow cytometry or allele-specific oligonucleotide PCR (with a sensitivity of one CLL cell per 10,000 leukocytes) and can be performed on peripheral blood samples.^35^ MRD testing is currently employed primarily for patients treated on clinical trials, but as future treatment and assay development progresses it may became a useful tool in monitoring of treatment efficacy.

## Conclusions:

The advent of novel agents with activity in CLL, including antibodies such as alemtuzumab,^37^ anti-CD20 ofatumumab, and anti-CD23 lumiliximab; BCL2 inhibitors such as oblimersen and ABT263; immunomodulatory drug lenalidimide; and cyclin-dependent kinase inhibitor flavopiridol, may change the paradigms in CLL therapy. Several of these agents show strong indications of activity in CLL, although some are still in clinical trials. Thus far, the fact remains that for poor-risk CLL, alloHCT is the only treatment with the potential of providing long-term disease control. Future combinations with emerging low-toxicity therapies may further enhance the curative potential of allogeniec hematopoietic cell transplant. New drugs can also potentially enable refractory patients to attain response as a bridge to more effective stem cell transplantation.

It is important to emphasize that patients with refractory disease should be treated within clinical trials whenever possible. Although there is no doubt that alloHCT can improve the prognosis of selected poor-risk patients, its place in the treatment algorithm for the general population of CLL patients is still unclear. This question can be properly addressed only by prospective trials comparing alloHCT with non-transplant chemo-immunotherapy strategies. The German CLL Study Group is currently proposing a trial aiming at validation of the EBMT criteria in patients with high-risk and very high-risk CLL. This trial would give further guidance regarding when and how to use alloHCT in poor-risk CLL. Until then, the data from retrospective and prospective studies provided by the cooperative groups and large transplant centers, including City of Hope, remain valuable sources of information.
